# Protein Adsorption on Solid Supported Membranes: Monitoring the Transport Activity of P-Type ATPases

**DOI:** 10.3390/molecules25184167

**Published:** 2020-09-11

**Authors:** Francesco Tadini-Buoninsegni

**Affiliations:** Department of Chemistry “Ugo Schiff”, University of Florence, 50019 Sesto Fiorentino, Italy; francesco.tadini@unifi.it; Tel.: +39-055-4573239

**Keywords:** sarcoplasmic reticulum Ca^2+^-ATPase, Cu^+^-ATPase, phospholipid flippase, charge displacement, concentration jump, solid supported membrane, conformational transition, electrogenicity, ion translocation, phospholipid flipping

## Abstract

P-type ATPases are a large family of membrane transporters that are found in all forms of life. These enzymes couple ATP hydrolysis to the transport of various ions or phospholipids across cellular membranes, thereby generating and maintaining crucial electrochemical potential gradients. P-type ATPases have been studied by a variety of methods that have provided a wealth of information about the structure, function, and regulation of this class of enzymes. Among the many techniques used to investigate P-type ATPases, the electrical method based on solid supported membranes (SSM) was employed to investigate the transport mechanism of various ion pumps. In particular, the SSM method allows the direct measurement of charge movements generated by the ATPase following adsorption of the membrane-bound enzyme on the SSM surface and chemical activation by a substrate concentration jump. This kind of measurement was useful to identify electrogenic partial reactions and localize ion translocation in the reaction cycle of the membrane transporter. In the present review, we discuss how the SSM method has contributed to investigate some key features of the transport mechanism of P-type ATPases, with a special focus on sarcoplasmic reticulum Ca^2+^-ATPase, mammalian Cu^+^-ATPases (ATP7A and ATP7B), and phospholipid flippase ATP8A2.

## 1. Introduction

P-type ATPases constitute a superfamily of membrane transporters that are present in all forms of life and are located in various membrane types, such as the plasma or cellular organelle membranes. The superfamily of P-type ATPases is classified into five distinct subfamilies (P1–P5), which are specific to different substrates [1,2,3]. These enzymes use the energy provided by ATP hydrolysis to transport various ions or phospholipids across cellular membranes, thereby generating and maintaining essential electrochemical potential gradients.

P-type ATPases share a similar molecular architecture, which comprises three distinct cytosolic domains, i.e., the actuator (A), nucleotide binding (N) and phosphorylation (P) domains, and two transmembrane domains, the transport domain of six helical segments (TM1 to TM6), which contains the ion binding sites located halfway through the membrane, and a class-specific support domain of four helical segments (TM7 to TM10). Moreover, in many P-type ATPases, the N- or C-terminal extensions at the cytosolic side act as regulatory (R) domains, which are autoinhibitory or can function as sensors for the transported cations [3,4]. Interestingly, the R domains of P-type ATPases have the characteristics of disordered proteins and are therefore highly variable and flexible. The disordered structure of the R domains is likely to facilitate their regulatory function favoring interaction with binding partners and helping to stabilize particular enzyme conformations [4,5].

P-type ATPases couple ion transport and ATP hydrolysis in a cyclic sequence of partial reactions that constitute the catalytic cycle. During catalysis, a transient phosphorylated intermediate is formed by the interaction of ATP with a conserved aspartate residue in the P domain, which is a specific feature of P-type ATPases. The Albers–Post or E_1_–E_2_ scheme [6,7] is the generally accepted model of the catalytic cycle of P-type ATPases. According to this model, the ATPase protein can assume two main conformational states, denoted E_1_ and E_2_, with different affinity for the transported ions and accessibility of the ion binding sites to the cytoplasmic and extracellular/luminal side. During the catalytic cycle, the ATPase undergoes structural rearrangements and conformational transitions between E_1_ and E_2_ states to perform ATP-driven transport of ions or phospholipids across the membrane [3,4].

**Figure 1:**
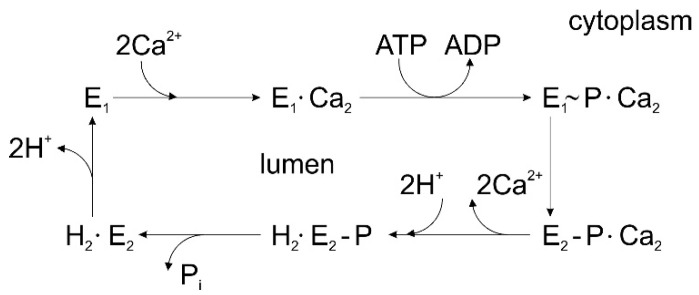
Simplified diagram of sequential reactions in the transport cycle of sarcoplasmic reticulum Ca^2+^-ATPase (SERCA).

The molecular mechanism of transport by P-type ATPases has been described in several reviews, see e.g., [1,3,4,8,9,10]. Figure 1 shows a simplified diagram of sequential reactions in the catalytic cycle of sarcoplasmic reticulum Ca^2+^-ATPase (SERCA) [11]. Starting at the E_1_ conformation, the SERCA cycle includes initial enzyme activation by high-affinity binding of two Ca^2+^ ions from the cytoplasmic side, followed by enzyme phosphorylation by ATP and the formation of a high-energy phosphorylated state E_1_~P (an ADP-sensitive phosphorylated intermediate that retains sufficient chemical energy to be able to transfer the phosphate to ADP, thus forming ATP). A conformational transition from the E_1_~P state to the lower energy phosphoenzyme intermediate E_2_P (an ADP-insensitive phosphorylated intermediate whose relatively low energy is suggested by its non-reactivity with ADP) favors the translocation of Ca^2+^ ions across the membrane and their release into the sarcoplasmic reticulum (SR) lumen in exchange for two luminal protons. Hydrolytic cleavage of the phosphoenzyme E_2_P (dephosphorylation) is followed by proton translocation and release to the cytosolic side, thus accelerating the E_2_ to E_1_ conformational transition, which completes the catalytic and transport cycle. Following the first high-resolution crystal structure of SERCA with bound Ca^2+^ (i.e., the E_1_·Ca_2_ state) [12], several crystal structures of SERCA in different conformational states in the transport cycle have been determined at atomic resolution, as reviewed in e.g., [10,13,14,15,16,17,18,19].

An electrophysiological method based on solid supported membranes (SSMs) has been used successfully to monitor the transport of charged substrates in various membrane transporters, including P-type ATPases [20]. SSM measurements of electrical currents can provide mechanistic and kinetic information about the movement of charged substrates within the membrane transporter as well as about conformational transitions associated with charge transfer in the reaction cycle of the membrane transport protein. In the present review, we will present the main features of the SSM-based electrophysiological method and discuss how the technique has contributed to investigating key aspects of the transport mechanism of P-type ATPases, with a special focus on SERCA, mammalian Cu^+^-ATPases, and a phospholipid flippase.

## 2. Current Measurements on Solid Supported Membranes

**Figure 2:**
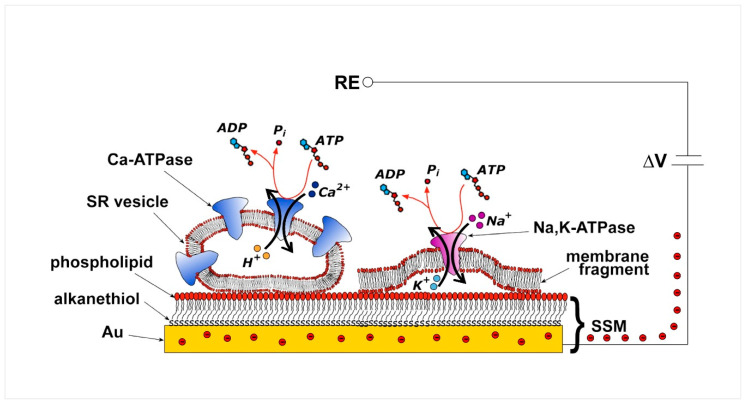
Schematic diagram of an sarcoplasmic reticulum (SR) vesicle containing Ca^2+^-ATPase and of a membrane fragment incorporating Na^+^,K^+^-ATPase adsorbed on a solid supported membranes (SSM) and subjected to an ATP concentration jump. If the ATP jump induces charge movement across the ATPase, a compensating electrical current flows along the external circuit (the red spheres represent electrons) if the potential difference (ΔV) applied across the whole system is kept constant. RE, reference electrode. Adapted with permission from [23]. Copyright 2009 American Chemical Society.

The SSM represents a convenient model system for a lipid bilayer membrane. In particular, the SSM consists of a hybrid alkanethiol/phospholipid bilayer supported by a gold electrode. The SSM is formed by covering the gold surface with an alkanethiol monolayer, usually an octadecanethiol monolayer, and then by self-assembling a phospholipid monolayer on top of the gold-supported thiol layer [21,22]. The so-formed hybrid bilayer (Figure 2) is characterized by a high mechanical stability so that fast solution exchange can be performed at the SSM surface. The exchange of solutions provides the substrate or ligand and activates the membrane transporter adsorbed on the SSM [22].

Various membrane preparations containing the transport protein of interest, i.e., native membrane vesicles, purified membrane fragments, and proteoliposomes with reconstituted proteins, can be physically adsorbed on the SSM (Figure 2). Adsorption of such membrane preparations allows a variety of transport proteins to be immobilized on the SSM surface in a simple spontaneous process. This experimental approach is much easier and more effective than direct incorporation of the membrane transporter in a free-standing planar lipid bilayer, such as the black lipid membrane, which requires complicated incorporation procedures, leading to a superior signal-to-noise ratio and time resolution of the electrical measurement.

Following stable adsorption of the membrane sample on the SSM, the membrane transporter is subjected to a substrate concentration jump through the solution exchange technique. A rapid exchange from a solution with no substrate for the membrane transporter to one containing a specific substrate, e.g., ATP for P-type ATPases, activates the transport protein. If the substrate concentration jump induces charge displacement across the protein, an electrical current is measured due to capacitive coupling between the membrane sample and the SSM [20,24,25]. In particular, movement of a net charge across the activated protein is compensated by a flow of electrons along the external circuit toward the electrode surface, to keep constant the potential difference (ΔV) applied across the whole metal/solution interphase (Figure 2) [20]. This flow of electrons corresponds to the measured capacitive current, which is strictly correlated with the transporter-generated current and is recorded as a transient current signal [20,24,25]. The SSM method allows the measurement of charge displacement under pre-steady state conditions, while steady-state currents are not recorded. We point out that the electrical behavior of the system is essentially the same whether membrane vesicles or membrane fragments are adsorbed on the SSM.

The transport mechanism of various P-type ATPases belonging to different subfamilies was characterized using the SSM technique, such as in the case of Na^+^,K^+^-ATPase [22,26], SERCA [27,28], and H^+^,K^+^-ATPase [29], belonging to the P2-ATPase subfamily, and more recently bacterial and mammalian Cu^+^-ATPases of subclass P1B [30,31] and P4-ATPase phospholipid flippase [32]. On the other hand, the P3-ATPase subfamily, which comprises plasma membrane H^+^-ATPases of fungal and plant cells, has not yet been investigated by the SSM method.

SSM measurements on P-type ATPases were useful to identify electrogenic steps, i.e., reaction steps associated with a net charge transfer, and to assign time constants to partial reactions in the ATPase transport cycle. However, slow transport processes with time constants greater than 200 ms can be hardly recorded in SSM-based current measurements [20].

Finally, the SSM technique has been successfully employed to evaluate the effects of pharmacologically relevant compounds, such as anti-cancer drugs [33], on the transport activity of P-type ATPases and to characterize the interaction of specific ATPase inhibitors, thereby providing a quantitative estimate of inhibition potency (IC_50_ values).

Analysis systems for SSM-based electrophysiology are commercially available and are based on the SURFE^2^R (Surface Electrogenic Event Reader) technology, as described in [29,34,35,36]. When higher throughput is required as in the case of drug screening, a fully automated device allows measuring electrical currents simultaneously from 96 individual SSM sensors in a parallel mode.

## 3. P-Type ATPases Investigated on Solid Supported Membranes

As mentioned above, the SSM technique was used to investigate net charge translocation (electrogenic transport) in P-type ATPases. Charge displacement associated with specific steps, i.e., ion binding/release, ion translocation, and exchange was measured in the ATPase transport cycle and the electrogenicity of partial reactions was determined, thereby providing mechanistic insights in the transport mechanism of different P-type ATPases. For example, a direct proof for the electrogenicity of cytoplasmic Na^+^ binding to the Na^+^,K^+^-ATPase was obtained with Na^+^ concentration jump experiments performed on membrane fragments containing Na^+^,K^+^-ATPase adsorbed on the SSM [26]. It was found that the charge associated with the Na^+^ binding step is about 30% of the displaced charge related to Na^+^ translocation and release, indicating that cytoplasmic Na^+^ binding is a minor electrogenic event in the reaction cycle of Na^+^,K^+^-ATPase [26].

In the next sections, we will discuss the contribution of the SSM technique to unravel key features of the electrogenic transport activity of some prominent members of the P-type ATPase family. In particular, the focus of the present review is on SERCA, Cu^+^-ATPases ATP7A and ATP7B, and P4-ATPase (phospholipid flippase) ATP8A2. SERCA has been characterized in detail by the SSM technique, providing useful information on the enzyme’s transport mechanism. This information was used for a comparative analysis of the transport properties of the Cu^+^-ATPases and phospholipid flippase, which were recently investigated by the SSM method.

### 3.1. Sarcoplasmic Reticulum Ca^2+^-ATPase

The SERCA enzyme is one of the most investigated P-type ATPase [15,16,37,38,39]. In muscle cells, SERCA couples the energy gained by the hydrolysis of one ATP molecule to the transport of two Ca^2+^ ions against their electrochemical potential gradient from the cytoplasm into the lumen of SR, which is the main intracellular Ca^2+^ storage organelle. Ca^2+^ uptake in the SR lumen by SERCA plays an essential role in regulating cytoplasmic Ca^2+^ concentration, which is kept at or below 0.1 µM; in this manner, SERCA induces muscle relaxation and contributes to intracellular Ca^2+^ homeostasis. Modified SERCA expression and impaired pumping activity have been associated with pathological conditions and several diseases with a wide range of severity [39,40].

SERCA (approximately 110 KDa) belongs to the P2A-ATPase subfamily. In mammals, SERCA is encoded by three different genes, ATP2A1-3, but isoform diversity is increased by alternative splicing of the transcripts, which raises the number of possible SERCA isoforms to more than 10 [41,42]. A very convenient experimental system for functional and structural studies of SERCA is provided by vesicular fragments of longitudinal SR, where SERCA1a is the predominant isoform. SR vesicles contain a high amount of SERCA, which accounts for approximately 50% of the total protein and which reaches a density in the SR membrane of about 30,000 µm^−2^ [43].

**Figure 3:**
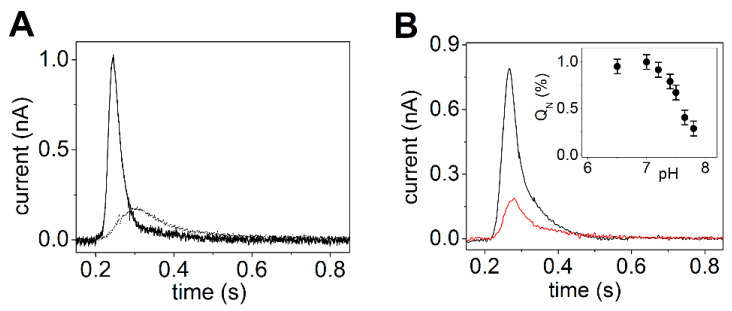
Transient currents generated by SERCA adsorbed on an SSM. (**A**) Transient current after a 10 µM free Ca^2+^ concentration jump in the absence of ATP (dotted line) and a 100 µM ATP concentration jump in the presence of 10 µM free Ca^2+^ (solid line). Reprinted from [44] with permission. (**B**) Current signals after 100 µM ATP concentration jumps in the presence of 10 µM free Ca^2+^ and 100 mM KCl at pH 7 (black line) and 7.8 (red line). The inset shows the dependence of the normalized charge (Q_N_) after 100 µM ATP concentration jumps on pH. The charges were normalized with respect to the maximum charge measured at pH 7. S.E. are given by error bars. Adapted from [48].

Electrical currents generated by SERCA were measured by adsorbing native SR vesicles containing SERCA1a from rabbit skeletal muscle on the SSM and by activating the calcium pumps with substrate, i.e., Ca^2+^ and ATP concentration jumps. The observed current signals allow the direct measurement of charge translocation by SERCA under different activation conditions. In particular, charge movements related to different electrogenic partial reactions in the SERCA transport cycle were detected. It was shown that a Ca^2+^ concentration jump in the absence of ATP induces a transient current (dotted line in Figure 3A), which is associated with an electrogenic event corresponding to enzyme activation by the initial binding of Ca^2+^ to the cytoplasmic side of the ATPase (the exterior of the SR vesicle, see Figure 2) [27,28,44]. When an ATP concentration jump was performed in the presence of Ca^2+^ ions, a current signal was detected (solid line in Figure 3A), which is associated with a further electrogenic step corresponding to ATP-dependent calcium translocation by the enzyme [20,27]. In particular, ATP concentration jump experiments on SR vesicles in the presence and absence of a calcium ionophore at different pH values [27] indicated that the ATP-induced electrical current is related to displacement and release of pre-bound Ca^2+^ at the luminal side of the pump (the interior of the SR vesicle, see Figure 2) after phosphorylation of the enzyme by ATP. The transient currents measured after a Ca^2+^ jump in the absence of ATP and an ATP jump in the presence of Ca^2+^ were both fully suppressed by thapsigargin [44], which is a highly specific and potent SERCA inhibitor [45,46]. We point out that to perform ATP hydrolysis and active Ca^2+^ transport SERCA undergoes large domain movements enabled by dynamic fluctuations and conformational transitions that are not random but instead are driven by the availability of specific substrates [47].

It is interesting to observe that the amplitude of the signal related to ATP-dependent Ca^2+^ translocation decreases as the pH is raised from 7 to 8 (Figure 3B). It is known that exchange of Ca^2+^ with H^+^ is a specific feature of SERCA [37,48], which favors Ca^2+^ release at the luminal side [17,49]. Useful information was provided by previous measurements on reconstituted proteoliposomes containing SERCA [49,50,51,52]. In particular, it was shown that the stoichiometry of the Ca^2+^/H^+^ countertransport is about 1/1 when the luminal and medium pH is near neutrality [49,52]. The importance of Ca^2+^/H^+^ exchange in determining the release of bound Ca^2+^ from the phosphoenzyme E_2_P was demonstrated in steady-state experiments on native SR vesicles [37]. It was reported that the maximal levels of accumulated Ca^2+^ are significantly reduced if the pH is raised above 7. This result shows that if exchange is limited due to low H^+^ concentration, Ca^2+^ is less likely to dissociate from the phosphoenzyme. Thus, the pH dependence of the current signals obtained with ATP concentration jumps (inset of Figure 3B) also indicates that when a lack of H^+^ limits Ca^2+^/H^+^ exchange, i.e., alkaline pH, the translocation of bound Ca^2+^ is prevented, even though K^+^ is present in high concentration and may neutralize acid residues at alkaline pH [48]. This suggests a requirement for specific H^+^ binding at the Ca^2+^ transport sites in order to obtain Ca^2+^ release.

The SSM method has also been used to investigate a very interesting research topic, which is currently receiving much attention, i.e., the molecular mechanisms of SERCA regulation. In muscle cells, SERCA transport activity is regulated by two analogous transmembrane proteins: phospholamban (PLN, 52 amino acids), which is primarily expressed in cardiac muscle where it regulates the SERCA2a isoform [53], and sarcolipin (SLN, 31 amino acids), which is mainly expressed in skeletal muscle where it regulates the SERCA1a isoform [54]. In particular, PLN inhibits pump activity by lowering the apparent Ca^2+^ affinity of SERCA, and the phosphorylation of PLN by protein kinases relieves SERCA inhibition [53]. There is general consensus that the PLN inhibition of SERCA involves the reversible physical interaction of a PLN monomer under calcium-free conditions. However, experimental evidence was provided that a PLN pentamer, which has been described as an inactive storage form, can also interact with SERCA [55,56].

**Figure 4:**
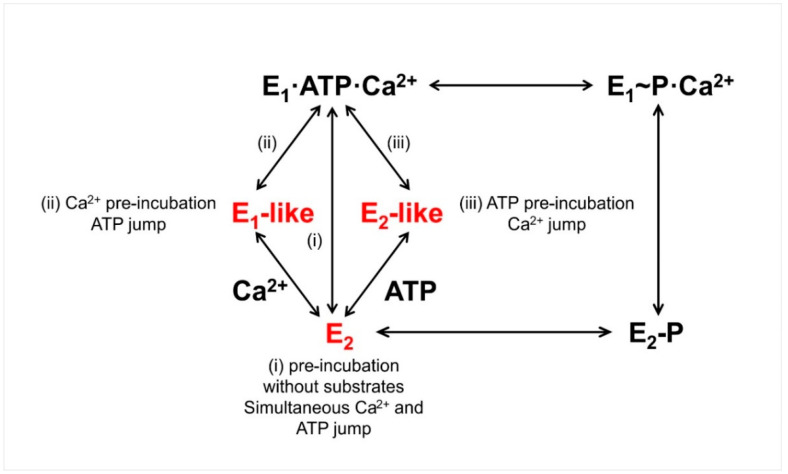
The SERCA transport cycle with relevant conformational states. The pre-incubation and concentration jump conditions used [57] are indicated. Shown in red are the calcium-free E_2_ state, an E_1_-like state promoted by calcium, and an E_2_-like state promoted by ATP. Adapted from [57].

To investigate the PLN effect on ATP-dependent Ca^2+^ translocation by SERCA, SSM-based current measurements were carried out on co-reconstituted proteoliposomes containing SERCA and PLN [57]. The proteoliposomes were adsorbed on the SSM and activated by Ca^2+^ and/or ATP concentration jumps. In particular, substrate conditions (various Ca^2+^ and ATP concentrations) were chosen that promoted specific conformational states of SERCA, from which calcium transport could be initiated. The results from pre-steady state charge (calcium) translocation experiments were compared with steady-state measurements of ATPase hydrolytic activity. It was found that the PLN effect on SERCA transport activity depends on substrate conditions, and PLN can establish an inhibitory interaction with multiple conformational states of SERCA (a calcium-free E_2_ state, a E_1_-like state promoted by Ca^2+^, and a E_2_-like state promoted by ATP, shown in red in Figure 4) with distinct effects on SERCA’s kinetic properties [57]. It was also noted that once a particular SERCA–PLN inhibitory interaction is established, it remains throughout the SERCA transport and catalytic cycle. These findings were interpreted on the basis of a conformational memory [58,59] in the interaction of PLN with SERCA, whereby a defined structural state of the SERCA/PLN regulatory complex, which depends on substrate conditions, is retained during SERCA turnover and conformational cycling.

In addition to PLN and SLN, single-span transmembrane proteins have recently been discovered that act as regulators of SERCA activity: dwarf open reading frame (DWORF), myoregulin (MLN), endoregulin (ELN), and another-regulin (ALN) [60,61,62]. While MLN, ELN, and ALN have been identified as inhibitors of SERCA activity, it was shown that DWORF does not inhibit the SERCA pump [62], enhancing Ca^2+^ uptake by displacing PLN. The oligomerization of these new SERCA regulators and the binding interaction of the monomeric form with the calcium pump were very recently investigated [63], thus providing a useful contribution in the characterization of the complexity of SERCA regulatory mechanisms. In this respect, it appears that the above-mentioned transmembrane peptides could be conveniently investigated by the SSM technique upon their reconstitution in proteoliposomes containing SERCA. This would help to elucidate the inhibitory or activation effects of the recently discovered SERCA regulators.

### 3.2. Cu^+^-ATPases ATP7A and ATP7B

The mammalian copper ATPases ATP7A and ATP7B are 165–170 KDa membrane proteins belonging to subclass IB of the P-type ATPase superfamily. At normal copper levels in the cell, ATP7A and ATP7B are found in the trans-Golgi network (TGN), and these enzymes translocate copper across the membrane from the cytoplasm into the TGN lumen using ATP hydrolysis [64,65,66,67,68]. ATP7A and ATP7B contribute to intracellular copper homeostasis by delivering copper to newly synthesized copper-containing proteins in the TGN and by removing copper excess from the cell [64]. ATP7A is expressed in most tissues but not in the liver, whereas ATP7B is mainly found in this organ [64]. The malfunction of either ATP7A or ATP7B is the cause of severe diseases, which are known as Menkes (ATP7A) and Wilson (ATP7B) diseases.

ATP7A and ATP7B show high sequence homology (about 60% identity). Their structure comprises eight transmembrane helices, which include a copper binding site (transmembrane metal binding site, TMBS), and the A, N and P cytoplasmic domains, which are common for P-type ATPases. A unique structural feature of ATP7A and ATP7B is the highly mobile N-terminal chain of six copper binding domains (N-terminal metal binding domain) that are involved in the copper-dependent regulation and intracellular localization of these enzymes [69].

As described in several reviews (e.g., [64,65,70,71,72,73,74]), Cu^+^ transfer by ATP7A and ATP7B involves copper acquisition from donor proteins on the cytoplasmic side of the membrane and copper delivery to acceptor proteins on the luminal side, without establishing a free Cu^+^ gradient. In conformity with other P-type ATPases, ATP7A and ATP7B hydrolyze ATP to form a transient phosphorylated intermediate, and they undergo conformational transitions that favor Cu^+^ transfer to/from the TMBS. From high-resolution crystal structures and molecular dynamics simulations on a bacterial Cu^+^-ATPase (*Legionella pneumophila* Cu^+^-ATPase, LpCopA) [75,76], it appears that copper ATPases have a unique copper release mechanism that is likely to be involved in specific and controlled Cu^+^ delivery to acceptor proteins.

**Figure 5:**
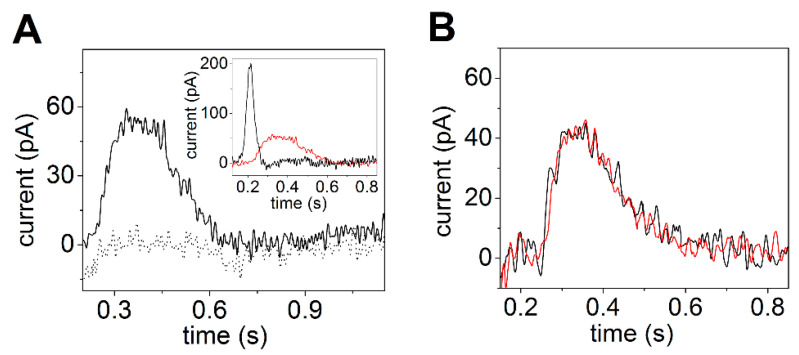
Transient currents generated by ATP7B adsorbed on an SSM. (**A**) Transient currents after 100 µM ATP concentration jumps in the presence of 5 µM CuCl_2_ (solid line) or 1 mM bathocuproinedisulfonate (BCS) (dotted line). The inset shows current signals after 100 µM ATP concentration jumps on ATP7B (red line) and SERCA (black line). Adapted from [31] with permission from Wiley. (**B**) Current signals after 100 µM ATP concentration jumps in the presence of 5 µM CuCl_2_ at pH 6 (black line) and 7.8 (red line). Adapted from [48].

Electrogenic copper movement within mammalian copper ATPases was demonstrated by current measurements on COS-1 microsomes expressing recombinant Cu^+^-ATPases (ATP7A and ATP7B) adsorbed on an SSM [31,48]. When an ATP concentration jump was performed on microsomes containing ATP7B (or ATP7A) in the presence of CuCl_2_ and dithiothreitol to reduce Cu^2+^ to Cu^+^, a current signal was obtained (solid line in Figure 5A), which was not observed when bathocuproinedisulfonate (BCS), acting as Cu^+^ chelator, was added to the reaction buffer (dotted line in Figure 5A). These experiments indicate that the copper-related current signal is associated with an electrogenic event corresponding to Cu^+^ movement within ATP7B upon phosphorylation by ATP [31,48], which is consistent with copper displacement from the TMBS to the luminal side of the enzyme.

By fitting the decay phase of the transient current with a first-order exponential decay function, a charge transfer decay time constant (τ) of 140 ms was determined for ATP7B, which is within the time frame of aspartate phosphorylation by ATP [31], suggesting that copper displacement in ATP7B is correlated to formation of the phosphorylated intermediate and precedes phosphoenzyme hydrolytic cleavage. This conclusion was also supported by SSM-based current measurements on the D1044A mutant of ATP7A. Asp1044 is the conserved aspartate residue in the P-domain of ATP7A that interacts with ATP to form the aspartyl phosphorylated intermediate. It was shown that the D1044A mutant yielded no current signal upon an ATP concentration jump in the presence of Cu^+^ [77]. This result further indicated that ATP-dependent copper movement through the ATPase is directly correlated to formation of the aspartyl phosphorylated intermediate by ATP consumption.

It is interesting to observe that ATP-induced copper movement in mammalian Cu^+^-ATPases is significantly slower than ATP-dependent Ca^2+^ translocation in SERCA [31], as shown by the different decay time constants τ for charge displacement following ATP jumps (inset of Figure 5A) on ATP7B (red line, τ = 140 ms) and SERCA (black line, τ = 25 ms). It is worth mentioning that the τ values for charge movements in ATP7B and SERCA are consistent with a slower phosphoenzyme formation in the copper ATPase [31] with respect to SERCA [78]. It should be noted that these decay time constants are attributed to initial partial reactions of the pump transport cycle and are not equivalent to steady-state turnover [31].

SSM measurements on ATP7A and ATP7B revealed that ATP-induced charge movement in these enzymes is not changed by alkaline or acid pH [48], as shown by charge transfer measurements at different pH values (Figure 5B). This finding indicated that copper displacement in ATP7A and ATP7B is pH independent, and it highlights a significant difference in the transport mechanisms of ATP7A/B and SERCA. It was proposed that in ATP7A/B, Cu^+^/H^+^ exchange may not be required for luminal copper release [48], as opposed to the strict requirement of Ca^2+^/H^+^ exchange in SERCA as discussed above. It is worth mentioning that carboxylate residues are absent in the ion-binding cluster located in the transmembrane region of the bacterial *Archaeoglobus fulgidus* CopA [79] and LpCopA [30], while crucial aspartate and glutamate residues are present in the equivalent transmembrane domain of SERCA [12,16,80] that are directly involved in Ca^2+^/H^+^ exchange. Thus, SSM measurements on ATP7A/B supported the hypothesis that Cu^+^ release in these enzymes may not be coupled to a net proton countertransport, which has not been observed for PIB-type ATPases [72,73,81]. Interestingly, a very recent study reported real-time fluorescence measurements on *E.coli* Cu^+^-ATPase (EcCopA) reconstituted in small unilamellar vesicles encapsulating a set of fluorescence probes that are selective for Cu^+^, pH, and membrane potential [82]. The results of this study demonstrated the absence of H^+^ countertransport in the Cu^+^ translocation cycle of EcCopA, qualifying EcCopA as an electrogenic uniporter.

### 3.3. P4-ATPase ATP8A2

A characteristic feature of eukaryotic cell membranes is the asymmetrical distribution of different lipids across the membrane bilayer. This is particularly evident in the plasma membrane, where phosphatidylcholine (PC) and sphingolipids, i.e., sphingomyelin and glycosphingolipids, are concentrated in the exoplasmic leaflet of the membrane, whereas phosphatidylserine (PS) and phosphatidylethanolamine (PE) are mainly restricted in the cytoplasmic leaflet [83,84,85,86]. Membrane lipid asymmetry is essential for a variety of cellular processes that include, e.g., cell and organelle shape determination, membrane stability and impermeability, membrane curvature, vesicle formation and trafficking, host–virus interactions, membrane protein regulation, blood coagulation, and apoptosis [86,87,88,89,90].

Phospholipid flippases, belonging to the P4-ATPase subfamily, couple ATP hydrolysis to the translocation of specific phospholipids from the exoplasmic to the cytoplasmic leaflet of biological membranes in order to generate and maintain transmembrane lipid asymmetry [89,91,92,93,94,95]. P4-ATPases are only found in eukaryotes and constitute the largest P-type ATPase subfamily. In mammals, at least 14 P4-ATPases are known, which are divided into five classes [89]. P4-ATPase dysfunction has been associated with severe neurological disorders and liver diseases in humans [92]. These lipid transporters consist of a large polypeptide with a molecular mass of about 120 kDa, which shares the general architecture of P-type ATPases. Most P4-ATPases form a heterodimeric complex with an accessory β-subunit of about 50 kDa belonging to the CDC50/LEM3 family [89,96,97]. High-resolution structures of yeast [98,99] and human [100] lipid flippases were determined by cryo-electron microscopy, as reviewed in [101], and very recently, the crystal structures of a human plasma membrane flippase were also reported [102].

**Figure 6:**
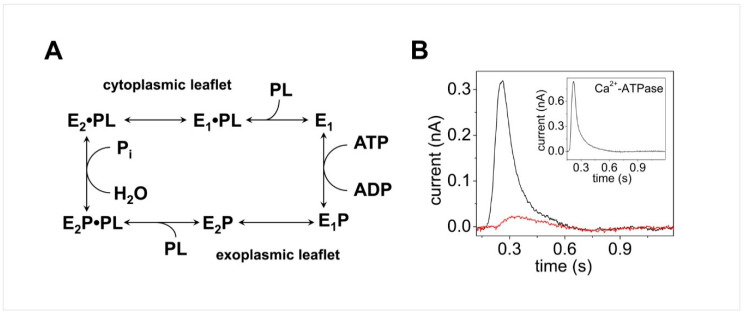
Simplified diagram of the ATP8A2 reaction cycle and transient currents generated by ATP8A2 adsorbed on a SSM. (**A**) E_1_, E_1_P, E_2_P, and E_2_ are different enzyme conformational states, where “P” indicates covalently bound phosphate. The phospholipid substrate, PL (phosphatidylserine (PS) or phosphatidylethanolamine (PE)), enters the cycle from the exoplasmic leaflet of the lipid bilayer by binding to the E_2_P phosphoenzyme, thereby stimulating the dephosphorylation and release of the lipid substrate toward the cytoplasmic leaflet as a consequence of the E_2_ to E_1_ conformational change. (**B**) Current transients observed upon 100 μM ATP concentration jumps on ATP8A2 reconstituted in proteoliposomes containing a mixture of 90% PC and 10% PS, in the absence (black line) or in the presence (red line) of 50 μM orthovanadate. The inset shows the current signal induced by a 100 μM ATP concentration jump on native SR vesicles containing SERCA. Reprinted from [32].

The transport mechanism of P4-ATPases is the subject of intensive research, and various models have been proposed for the phospholipid translocation pathway in P4-ATPases [103,104,105,106,107]. The recent atomic resolution structures of yeast and human P4-ATPases have provided valuable information on different conformational states in the flippase transport cycle, which is depicted by the Albers–Post or E_1_–E_2_ scheme commonly used to describe the mechanism of ion transporting P2-type ATPases. The P4-ATPase reaction cycle (see the simplified diagram in Figure 6A) has been examined in some detail for the mammalian flippase ATP8A2 [108], which is highly expressed in the retina, brain, testis, and spinal cord. It was shown that ATP8A2 is phosphorylated by ATP at the aspartate conserved in all P-type ATPases, and the phosphoenzyme exists in E_1_P and E_2_P states [108]. Dephosphorylation of the E_2_P state is activated by binding of the two known substrates PS and PE, but not by binding of PC that is not a substrate of ATP8A2 [109], and dephosphorylation is associated with lipid translocation from the exoplasmic to the cytoplasmic leaflet of the membrane bilayer. Although significant progress has been made in our understanding of phospholipid flipping by P4-ATPases, several aspects of the flippase transport mechanism remain to be explored, such as the stoichiometry of phospholipid molecules translocated per ATP hydrolyzed, the mechanisms underlying lipid binding and release, the electrogenicity of phospholipid transport, and the related issue of countertransport, i.e., countertransport of an ion or other charged substrate from the cytoplasm to the exoplasm in connection with the E_1_ → E_1_P → E_2_P reaction sequence as observed for P2-type ATPases.

To address unexplored key aspects of the flipping mechanism of P4-ATPases, in particular the electrogenicity of phospholipid flippases and ion countertransport, the SSM method was very recently used in a study of the elctrogenic properties of wild-type and mutant forms of the flippase ATP8A2 [32]. Purified ATP8A2 and its accessory CDC50A protein were reconstituted in proteoliposomes of different lipid compositions that were adsorbed on the SSM surface and subjected to ATP concentration jumps. It was shown that an ATP jump on ATP8A2 reconstituted into proteoliposomes consisting of a mixture of 90% PC and 10% PS induced a current signal (black line in Figure 6B) that was completely suppressed in the presence of the ATPase inhibitor orthovanadate (red line Figure 6B). Since orthovanadate binds to the ATPase from the cytoplasmic side, it was concluded that the ATPase molecules with the cytoplasmic side facing the external aqueous solution generated the ATP-dependent charge movement across ATP8A2. It was also noted that the sign of the ATP8A2-related current signal is positive, as observed for the SERCA-related transient current (inset of Figure 6B) that is attributed to the translocation and release of Ca^2+^ ions into the SR vesicle interior [27] (see Section 3.1). We point out that the movement of positive charge in one direction is electrically equivalent to the displacement of negative charge in the opposite direction. Therefore, the ATP8A2 current signal was associated with ATP-dependent translocation of the negatively charged PS toward the outside of the proteoliposomes (the ATP8A2 cytoplasmic side facing the external aqueous solution) [32].

It is worth noting that PC, also present in the proteoliposomes, is not a substrate for ATP8A2 and has an electrically neutral head group. As mentioned above, PC does not stimulate ATP8A2 dephosphorylation; however, the enzyme can be phosphorylated by ATP in the absence of PS and PE and in the presence of PC alone [108]. Interestingly, an ATP concentration jump on ATP8A2 reconstituted in proteoliposomes containing 100% PC yielded no significant transient current, indicating that enzyme phosphorylation by ATP (E_1_→E_1_P reaction step) does not generate any electrical signal [32].

Useful information was also provided by ATP concentration jump experiments on proteoliposomes containing selected mutants of ATP8A2 [32]. In particular, the E198Q mutation was examined. Glu-198 is located in the DGET motif of the cytoplasmic A-domain of ATP8A2 that facilitates dephosphorylation of the phosphorylated intermediate. It was reported that phosphorylation by ATP is allowed in E198Q, while dephosphorylation is blocked with resulting E_2_P accumulation [108]. The absence of an electrical current upon an ATP concentration jump on proteoliposomes containing E198Q indicated that the electrogenicity of ATP8A2 is not related to the E_1_→E_1_P step (phosphorylation by ATP) or with the E_1_P→E_2_P conformational transition of the enzyme, which is in agreement with the experiments on 100% PC proteoliposomes. This finding is important to address the issue of whether ion countertransport occurs from the cytoplasmic to the exoplasmic side of the phospholipid flippase. In fact, it was shown that no charged substrate is countertransported during the E_1_ → E_1_P → E_2_P reaction sequence [32], thereby distinguishing the P4-ATPase from the P2-type ATPases, which transport ions in opposite directions and are therefore referred to as antiporters.

It was concluded that the electrogenicity of ATP8A2 is related to a step in the ATPase transport cycle directly involved in translocation of the phospholipid [32]: dephosphorylation of the E_2_P intermediate, activated by lipid binding from the exoplasmic side, and/or the subsequent E_2_ → E_1_ conformational transition of the dephosphoenzyme, which is associated with release of the lipid to the cytoplasmic side [104]. It is noteworthy that the findings of the SSM study of the mammalian phospholipid flippase [32] support the view that the P4-ATPase is most likely an electrogenic uniporter, as also recently reported for a bacterial Cu^+^-transporting P1B-ATPase [82].

## 4. Conclusions

The charge transport mechanism of various P-type ATPases has been conveniently investigated by adsorbing the membrane-bound ATPase on the SSM and by activating the enzyme with a concentration jump of a suitable substrate. The transient current observed with the SSM method is a direct measurement of charge movements occurring during reactions involved in the transport cycle of the ATPase. This kind of measurements allows the identification of electrogenic partial reactions, which in turn can be used to localize charge translocation in the reaction cycle of the ion/lipid pump. It was shown that charge displacement in P-type ATPases is associated with transitions between different conformational states that facilitate the binding or release of the charged substrate.

The SSM method can provide useful information about the transport activity of P-type ATPases, as well as the molecular mechanisms of ATPase regulation, as discussed in the case of the SERCA enzyme. This is actually a very interesting research topic, which has yet to be examined in detail for some P-type ATPases, such as mammalian P4-ATPases [89]. However, also for the well-characterized P2-type ATPases Na^+^,K^+^-ATPase, and SERCA, a complexity of regulatory mechanisms has emerged [4,19], which requires further detailed investigation. We think that the SSM method can be usefully employed to characterize the interaction of P-type ATPases with specific regulatory partners, which can be small molecules, soluble proteins, transmembrane peptides, or the surrounding lipid bilayer [110].

Another interesting field of application of the SSM technique is related to the drug discovery process. It has been shown that drug/protein interactions can be conveniently monitored on SSMs. In particular, the SSM technique has demonstrated its usefulness to investigate the effects of various pharmaceutically relevant compounds on P-type ATPases [29,33,111], which are important targets for a variety of drugs [112,113,114]. Therefore, this technique represents a robust and reliable assay in drug development and evaluation studies on membrane transport proteins, as it can be used to quantify the effectiveness and potency of drugs directed toward specific protein targets, and to characterize novel drug candidates.
